# Genome Sequence of a Moderately Halophilic Bacillus cereus Strain, TS2, Isolated from Saltern Sediments

**DOI:** 10.1128/MRA.00873-18

**Published:** 2018-08-16

**Authors:** Manoharan Shankar, Anbazhagan Mageswari, Chandrasekaran Suganthi, Paramasamy Gunasekaran, Kodiveri M. Gothandam, Sivashanmugam Karthikeyan

**Affiliations:** a School of Bio-Sciences and Technology, Vellore Institute of Technology, Vellore, Tamil Nadu, India; Georgia Institute of Technology

## Abstract

We report the 5.3-Mbp genome sequence of Bacillus cereus strain TS2, which was isolated from the sediments of a solar saltern in southern India. Genome analysis of B. cereus TS2, a salt-resistant strain, will improve our understanding of how B. cereus, a food pathogen, responds to hyperosmotic stress.

## ANNOUNCEMENT

Bacillus cereus is one of the most frequent causes of food contamination and consequent food poisoning. Owing to its ability to produce enterotoxins, B. cereus can cause food poisoning, which manifests as either an emetic or a diarrheal syndrome (1). This is achieved by means of evading the host’s defenses by production of various virulence factors that also allow the pathogen to damage the host’s intestinal epithelial cells, resulting in diarrhea (2). Since some B. cereus strains and spores are capable of surviving normal food preservation methods, including refrigeration (3), use of heat, alkali, or acid (4), and salt treatment (5), it is essential to study the mechanisms by which resistance to these treatments is achieved.

B. cereus TS2 was isolated from the sediments of a solar saltern in Tuticorin, Tamil Nadu, India, and was capable of growing in 8% NaCl. The sequence of the B. cereus TS2 genome was determined on a HiSeq 2000 sequencing system per the standard Illumina protocol. Paired-end sequence reads (100 bp) were subjected to adapter removal and quality control using the SeqQC v2.2 suite (Genotypic, Bangalore, India). SPAdes v3.1.0 (6) was then used for *de novo* assembly of the high-quality reads into 173 contigs (*N*_50_, 537,946 bp). Using the scaffolding tool SSPACE v2.0 (7), the 173 contigs were organized into 121 scaffolds (*N*_50_, 1,475,409 bp). Finally, using the B. cereus Q1 genome (NCBI accession no. NC_011969) as a reference, gap closure was attempted using GapCloser v1.12 from the SOAP*denovo*2 assembly package (8), resulting in 24 gap-joined scaffolds totaling 5,308,102 bp in length with a GC content of 35.3%.

Gene prediction using the Rapid Annotation using Subsystems Technology (RAST) server (9) led to the identification of 5,493 coding sequences. We identified homologs of the GroELS and DnaK heat shock response chaperonin families in B. cereus TS2, among other heat shock response-inducible genes. Strain TS2 also harbored the cold shock response proteins CspA and CspD, which allow Bacillus subtilis to survive at low food preservation temperatures (10). Homologs of genes previously implicated in Gram-positive salt stress response (11), including the K^+^ uptake protein TrkH, osmoprotectant uptake (Opu) class choline/glycine betaine uptake systems, and the l-proline transporter ProP, were identified on the TS2 chromosome. We also identified various virulence factors, including the toxins (enterotoxins A, B, and C, a predicted hemolysin, and cytotoxin K) (12) usually encoded by pathogenic B. cereus strains. Additionally, hydrolytic enzymes (phospholipase C, sphingomyelinase C, and collagenase), which act as virulence factors *in vivo*, were detected in B. cereus TS2. Homologs of acid resistance determinants (F1F0-ATPase, cyclopropane fatty acyl phospholipid synthase, and amino acid decarboxylases) which enable B. cereus to transit through the hostile gastric environment (13) and reach the intestines were also detected. To our surprise, we also detected resistance mechanisms against zinc, arsenic, cobalt, cadmium, copper, chromium, bile, and antibiotics (streptothricin, tetracycline, aminoglycosides, vancomycin, fosfomycin, fluoroquinolones, and β-lactam antibiotics) encoded on the TS2 genome. We are currently testing and evaluating B. cereus TS2 as a potential food contaminant and a pathogen.

### Data availability.

This whole-genome shotgun project has been deposited in DDBJ/ENA/GenBank under the accession no. PIYQ00000000. The version described in this paper is the first version, PIYQ01000000.
